# Submonolayer biolasers for ultrasensitive biomarker detection

**DOI:** 10.1038/s41377-023-01335-8

**Published:** 2023-12-06

**Authors:** Chaoyang Gong, Xi Yang, Shui-Jing Tang, Qian-Qian Zhang, Yanqiong Wang, Yi-Ling Liu, Yu-Cheng Chen, Gang-Ding Peng, Xudong Fan, Yun-Feng Xiao, Yun-Jiang Rao, Yuan Gong

**Affiliations:** 1 Key Laboratory of Optical Fiber Sensing and Communications (Ministry of Education of China), School of Information and Communication Engineering, University of Electronic Science and Technology of China, Chengdu, Sichuan 611731 China; 2 Key Laboratory of Optoelectronic Technology and Systems (Ministry of Education of China), School of Optoelectronic Engineering, Chongqing University, Chongqing, 400044 China; 3 State Key Laboratory for Mesoscopic Physics and Frontiers Science Centre for Nano-optoelectronics, School of Physics, Peking University, Beijing, 100871 China; 4 School of Electrical and Electronic Engineering, Nanyang Technological University, Singapore, 639798 Singapore; 5 School of Electrical Engineering and Telecommunications, University of New South Wales, Sydney, NSW 2052 Australia; 6 Department of Biomedical Engineering, University of Michigan, Ann Arbor, MI 48109 USA; 7 Research Centre for Optical Fiber Sensing, Zhejiang Laboratory, Hangzhou, Zhejiang 310000 China

**Keywords:** Imaging and sensing, Microresonators

## Abstract

Biomarker detection is key to identifying health risks. However, designing sensitive and single-use biosensors for early diagnosis remains a major challenge. Here, we report submonolayer lasers on optical fibers as ultrasensitive and disposable biosensors. Telecom optical fibers serve as distributed optical microcavities with high Q-factor, great repeatability, and ultralow cost, which enables whispering-gallery laser emission to detect biomarkers. It is found that the sensing performance strongly depends on the number of gain molecules. The submonolayer lasers obtained a six-order-of-magnitude improvement in the lower limit of detection (LOD) when compared to saturated monolayer lasers. We further achieve an ultrasensitive immunoassay for a Parkinson’s disease biomarker, alpha-synuclein (α-syn), with a lower LOD of 0.32 pM in serum, which is three orders of magnitude lower than the α-syn concentration in the serum of Parkinson’s disease patients. Our demonstration of submonolayer biolaser offers great potentials in high-throughput clinical diagnosis with ultimate sensitivity.

## Introduction

Early detection of diseases such as cancer and dementia before they manifest serious, irreversible symptoms is of considerable public health importance and can help reduce morbidity and mortality^1–3^. In the early stage of a disease, precisely estimating the extremely low concentrations of biomarkers is difficult^4–6^. Fluorescence and luminescence biosensors are highly active research fields due to their high sensitivity. The fluorescence biosensors usually suffer from influence of background noise, which deteriorates the sensing performance. The lluminescence biosensors have an extremely low background noise and are capable of pushing the limit of detection (LOD) down to the attomole level^7,8^. However, limited by the weak signal, chemiluminescence biosensors require highly sensitive detector like photomultiplier tubes. Optical biosensors, which amplify weak biological signals by enhancing light-matter interactions, are becoming a mainstream technology for sensitive biomarker detection^9–11^. To date, various types of optical methods based on interferometers^12,13^, surface plasmon resonance (SPR)^14,15^, surface-enhanced Raman scattering (SERS)^16,17^, and optical microcavities^18–21^ have been developed to break the lower LOD record. Their performance, however, highly relies on meticulous design and precise fabrication, making high-throughput production of disposable diagnostic devices challenging^22–24^. Due to the amplification effect, even a minor fabrication error can cause considerable deviations in test results and deteriorate the sensing performance in single use^25,26^. This is particularly the case with ultrasensitive biosensors. Micro- and nano-interferometers rely on precision micromachining facilities, such as femtosecond lasers^27^, focused ion beams^28^, or electron beams^29^. SPR biosensors require thin film deposition with precise thickness at the nanometre scale^30^. SERS signals strongly depend on the properties of nanoparticles and substrates^31^. Optical microcavities have evolved as a powerful platform for amplifying optical signals with strong cavity feedback over the last two decades^32–37^, and they have been widely used for biological analysis^18–21^. The strong dependence on delicate fabrication procedures and the essential coupling requirement, however, are highly undesirable for single-use biosensors.

**Fig. 1:**
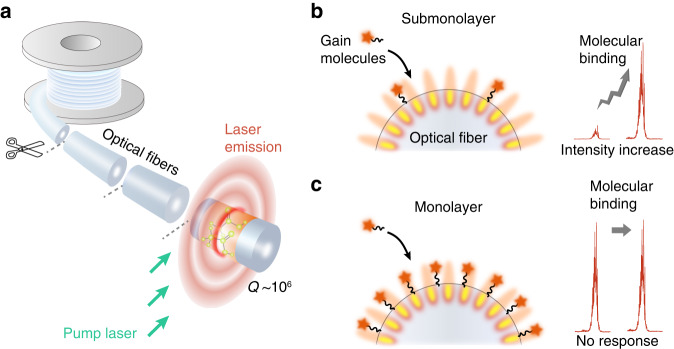
Schematic illustration of the submonolayer biolaser. **a** Submonolayer whispering-gallery biolasers on telecom optical fibers. **b**, **c** Conceptual illustration of submonolayer and monolayer biolasers. The intensity response of submonolayer biolaser shows a significant enhancement in sensitivity over the monolayer biolaser

Here, we propose the concept of submonolayer biolasers to bridge the gap between the sensitivity and single use of optical microcavities. The submonolayer biolasers were mass-produced at negligible cost using optical fiber microcavities that were distributed across an extraordinary length of 10 km and had ultrahigh Q-factors of 10^6^ (Fig. 1a). In striking contrast to passive microcavities, pumping and detection of submonolayer biolasers can be conveniently performed by free-space optics, which eliminates the dependence on critical waveguide coupling and, more importantly, enabling the development of single-use biosensors with ultrahigh sensitivity. By pushing the gain molecules down to the threshold density, the submonolayer biolaser demonstrates a six-order-of-magnitude improvement in LOD compared to the monolayer biolaser (Figs. 1b, c and 4). The submonolayer biolaser was further employed to detect a Parkinson’s disease (PD) biomarker in serum with a lower LOD of 0.32 pM. We envision that the single-use laser-based biosensors with ultrahigh sensitivity could enable cost-effective and early diagnosis of major diseases.

## Results

### Conceptual demonstration

**Fig. 2:**
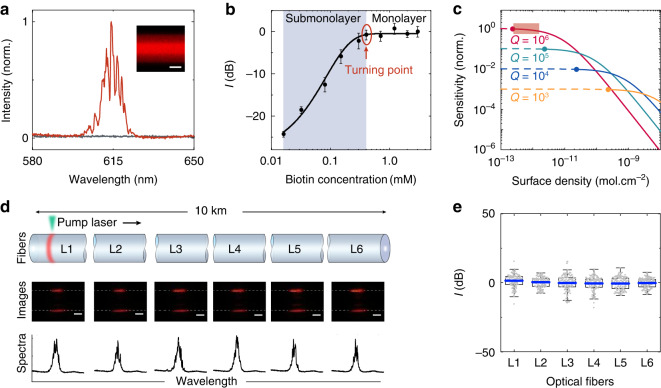
Characterization of the submonolayer biolasers. **a** Emission spectra. Red curve, pumping above the threshold; gray curve, pumping below the threshold. Inset, fluorescence image of the submonolayer biolaser. Scale bar, 50 μm. **b** Transition from the monolayer biolaser to the submonolayer biolaser by reducing the conjugation of biotin molecules. Each error bar is calculated from more than 60 lasers and denotes the 95% confidence interval. **c** Theoretical sensitivity as a function of the surface density of gain molecules. The solid dots denote the threshold molecular density for lasing, while the dashed curves correspond to the cases below the laser threshold. **d** Laser images and spectra of disposable submonolayer biolasers, with six segments of optical fibers (L1 to L6) randomly picked from a 10 km spool. Scale bar, 50 μm. **e** Statistical analysis of the laser intensity. The data were collected from 736 lasers on six segments of fibers. Blue bars, the average intensity of each fiber

We developed submonolayer biolasers based on sparse and specific molecular conjugation on optical fiber microcavities, and for the first time, we observed the transition from a monolayer biolaser to a submonolayer biolaser (Fig. 2). The commercial single mode optical fiber (SMF-28e, Corning) was employed for the conceptual demonstration of submonolayer biolasers. Multimode fiber and other types of commercial optical fibers are also suitable for this experiment. The optical fiber was biotinylated before being conjugated with streptavidin-Cy3 (Sav-Cy3) molecules that served as laser gain molecules (Fig. S1). The bright fluorescence image demonstrates that Cy3 molecules were successfully conjugated (inset of Fig. 2a). In an aqueous environment, the optical fiber microcavities have a high Q-factor surpassing 10^6^, allowing strong light-matter interactions on the surface (Fig. S2). Sharp peaks were observed in the emission spectrum of submonolayer biolaser (Fig. 2a) and the emission intensity shows a threshold behavior (Fig. S3). The irregular position of peaks is due to the limited resolution (~0.72 nm) of spectrometer. The linewidth narrowing near threshold confirms the generation of laser emission (Fig. S4). Compared to fiber taper coupling in passive microcavity sensors, the lateral pump and collection in free space significantly improve the reproducibility for disposable usage. Because of the localization of molecules on the fiber surface, the conjugated molecules are thinner than 1/10 of the evanescent wave penetration depth (Fig. S5)^38–41^. As a result, unlike the homogeneous gain solution employed in traditional optofluidic lasers^42,43^, all gain molecules in submonolayer biolasers can participate in lasing, allowing ultrahigh sensitivity and ultralow fluorescence background. The surface density of Cy3 molecules can be specifically adjusted by biotin, which substantially influences the laser output (Fig. S1). When the surface density is reduced from 1.8 × 10^−12 ^mol·cm^−2^ to 8.2 × 10^−13 ^mol·cm^−2^ (Point A to Point B in Fig. S6), the laser threshold rises from 0.09 mJ·mm^−2^ to 0.22 mJ·mm^−2^ (Fig. S3). The threshold is comparable to that of other biolasers and is suitable for biomedical research^44–46^. The surface density also influences the sensitivity of the submonolayer biolaser, which will be discussed in the next section.

We explored the ultimate lasing limit of the biolaser by reducing the surface density of gain molecules (Fig. 2b) according to the specific binding mechanism (Fig. S1). First, monolayer biolasers were demonstrated, and the laser emission remained stable when the biotin concentration was gradually reduced to 0.4 mM (the saturated zone in Fig. 2b). Because residual molecules were washed away to enable specific biomolecular attachment, the upper limit is a saturated monolayer. With a biotin concentration of ~0.4 mM, a turning point indicating a state transition from a monolayer to a submonolayer biolaser is observed. The surface density at this turning point is ~1.8 × 10^−12 ^mol·cm^−2^, according to the calculations in Fig. S6. Following that, the laser intensity linearly declines with biotin on a log-log scale, defining the range of the submonolayer biolaser (the gradient zone in Fig. 2b).

### Disposable submonolayer biolasers

Currently, advanced fiber draw tower technology allows highly accurate control of the fiber geometry, including cladding diameter and non-circularity, and permits cost-effective manufacturing of more than 50,000 meters of optical fiber with a single preform. Silica optical fibers can be regarded as a series of distributed cylindrical optical microcavities supporting whispering-gallery modes (WGMs). Optical fibers are an attractive platform for disposable use due to their great repeatability and ease of fabrication at an ultralow cost.

We demonstrated disposable submonolayer biolasers made from different optical fiber segments randomly selected from a 10 km spool. The laser threshold is less than 1 mJ·mm^−2^, which is within the acceptable range for biological materials (Fig. S7). We then evaluated 736 biolasers distributed on six optical fiber segments (L1 to L6) by linearly scanning the pump laser while concurrently capturing the laser patterns and spectra (Fig. 2d). Due to the consistent geometry and surface properties of the optical fibers, the laser patterns and spectra obtained from different biolasers are similar. To quantify the laser emission of the submonolayer biolasers, we calculated the relative laser intensity in decibels (dB) (see Materials and methods for details), which agrees well with a normal distribution (Fig. S8). The pump laser was scanned along the optical fiber with a step of 250 μm, and each submonolayer biolaser at the pump location was regarded as a sensing element for disposable use. A considerable number of submonolayer biolasers can be measured in a short period of time, allowing statistical analysis of submonolayer biolasers. We used the average laser intensity as the sensing indicator throughout the experiment, with a variance of only 2% (Fig. 2e). This study validates the feasibility of realizing disposable submonolayer biolasers. A total of 5163 biolasers were employed as disposable sensing elements for threshold tests, conceptual demonstration, and biomarker detection. The disposable submonolayer biolasers are intrinsically safe and free of recalibration. They also allow parallel and high-throughput tests.

### Sensitivity analysis

We established a theoretical model (Eqs. S1 to S9) and calculated the sensitivity to demonstrate the sensing mechanism of the submonolayer biolasers (Fig. 2c)^47^. The numerical findings show that the sensitivity increases with lower surface density and higher Q-factor. When the surface density is reduced to below the turning point (1.8 × 10^−12 ^mol·cm^−2^, Fig. 2b), an ultrahigh sensitivity is observed due to the high Q-factor exceeding 10^6^. The linear fluorescence emission, in contrast, shows no improvement in the sensitivity with fewer fluorescent molecules (Eqs. S10 and S11). This phenomenon enables us to achieve an ultrahigh sensitivity by decreasing the surface density of gain molecules.

### Exploring the ultimate LOD

**Fig. 3:**
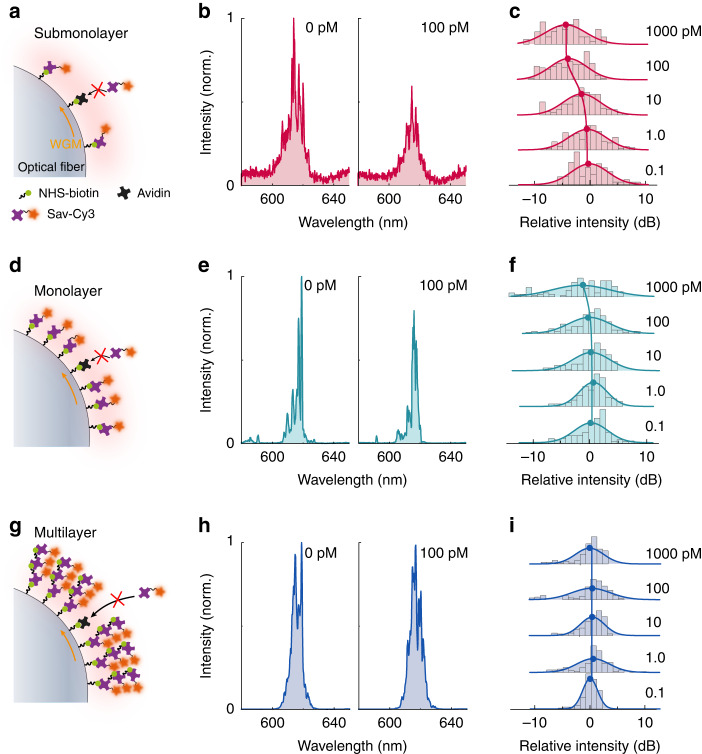
The intensity responses of biolasers to avidin. Conceptual illustrations of the submonolayer biolaser (**a**), monolayer biolaser (**d**), and multilayer biolaser (**g**). Comparison of the spectra (**b**, **e**, **h**) of the three types of biolasers with 0 pM and 100 pM avidin. **c**, **f**, **i** Dependence of the statistical distribution of the laser intensity on the avidin concentration

We experimentally demonstrated an ultrasensitive laser biosensor by reducing the surface density of gain molecules to the submonolayer level. We fabricated three types of biolasers on an optical fiber, i.e., submonolayer (Fig. 3a), monolayer (Fig. 3d), and multilayer (Fig. 3g) biolasers. The submonolayer and monolayer biolasers were fabricated with similar protocols with different biotin concentrations, while the multilayer biolasers were fabricated by assembling multilayers of Cy3 molecules using streptavidin bridging molecules. The detailed procedure is described in the Materials and methods.

We further characterized the LOD of the biolasers by employing the avidin-biotin interaction as an example due to its excellent affinity and specificity. This specific molecular conjugation has been widely exploited in mainstream clinical methods, including western blotting (WB)^48^ and enzyme-linked immunosorbent assay (ELISA)^49^. Please note that the avidin solution was incubated prior to the Sav-Cy3 to eliminate the influence of steric hindrance on avidin conjugation. With a higher avidin concentration, more binding sites (biotin) are occupied by avidin, resulting in fewer conjugated Sav-Cy3 molecules and a lower laser intensity. The submonolayer biolaser shows ultrahigh sensitivity owing to a considerable reduction in gain molecules. A dramatic decrease in the laser intensity (~40%) is observed when the avidin concentration is increased from 0 pM to 100 pM (Fig. 3b). The decline in the laser intensity can also be recognized in the statistical distribution, which shifts towards a lower intensity when the avidin concentration exceeds 10 pM (Fig. 3c). The intensity of the monolayer biolasers (Fig. 3d) falls slightly (7%) at 100 pM avidin (Fig. 3e), and a horizontal shift in the statistical distribution can be seen only at 1000 pM (Fig. 3f). Due to the relatively large number of gain molecules on the fiber in the multilayer biolasers (Fig. 3g), no observable changes in the laser spectra or the statistical distribution (Fig. 3h, i) are observed. These results indicate a significant enhancement of the sensitivity in the submonolayer biolasers.

**Fig. 4:**
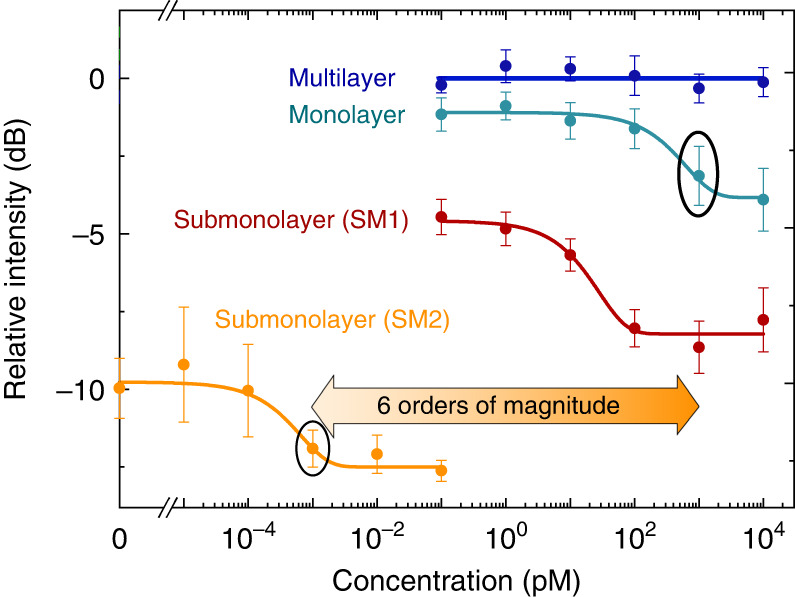
Comparison of the sensing performance of four types of biolasers. SM1 denotes the submonolayer biolaser in Fig. 3a–c, and SM2 denotes the submonolayer biolaser with gain molecules down to the threshold density

We investigated the ultimate LOD by reducing the gain molecules to the threshold density (~3.2 × 10^−13 ^mol·cm^−2^, Point C in Fig. S6). We recorded the laser intensity when gradually increasing the avidin concentration from the aM to fM level. A step change in the laser emission between 100 aM and 1 fM can be distinguished (yellow curve in Figs. 4 and S9). Compared to the monolayer biolaser, a six-order-of-magnitude improvement in the LOD are experimentally demonstrated in submonolayer biolaser (Fig. 4), indicating a greater potential in biomarker detection. This experiment was designed to highlight the substantial dependence of the LOD on the number of gain molecules. The lasing intensity had a negative sensitivity with the avidin concentration. This unique design substantially simplifies the experimental procedures to change the amount of conjugated gain molecules, but it is not optimized for a large sensing dynamic range. A narrow dynamic range around one order of magnitude was obtained in SM2 due to the threshold condition, which requires sufficient gain molecules for lasing.

### Detection of a Parkinson’s disease biomarker

**Fig. 5:**
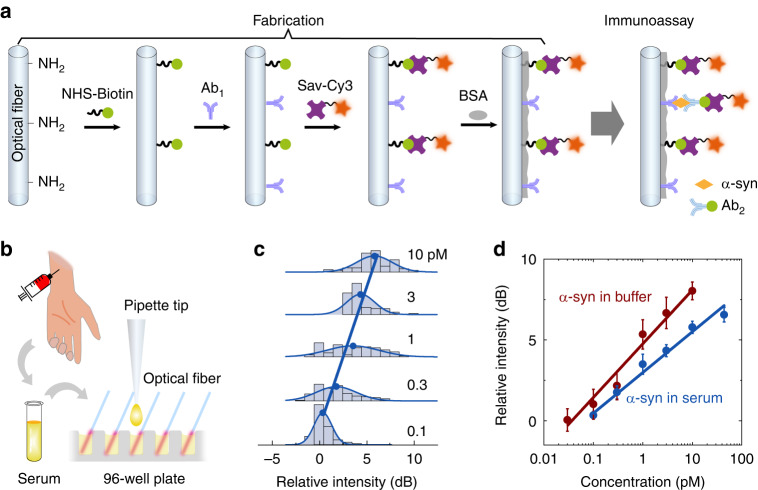
Alpha-synuclein detection by the submonolayer biolaser. **a** Conceptual illustration of the bioconjugation protocol for the submonolayer biolaser immunosensor. **b** Schematic diagram of the mass and disposable immunoassay in serum. **c** Statistical distribution of the relative intensity at different α-syn concentrations in serum. **d** Calibration curves for α-syn detection in buffer (red) and serum (blue). Each error bar is calculated from ~60 lasers and denotes the 95% confidence interval

Parkinson’s disease is one of the most frequent neurodegenerative disorders of the elderly and has affected more than 10 million people worldwide^50^. The pathogenesis of PD is strongly linked to a presynaptic neuronal protein called alpha-synuclein (α-syn)^51,52^. According to recent studies, the α-syn in cerebrospinal fluid and in serum can be employed as a PD biomarker^53^. The submonolayer biolasers were designed to detect α-syn with ultrahigh sensitivity, which offers great potentials for early-stage diagnostics (Fig. 5a).

Antigen molecules (α-syn) were sandwiched between capture antibodies (Ab_1_) and detection antibodies (Ab_2_). Because the immuno-relevant Sav-Cy3 was conjugated to biotin-labeled detection antibodies, a higher laser intensity reflects more biomarker molecules. The procedure of integrating the sandwiched immunoreaction into the submonolayer biolaser can be found in the Materials and methods section.

First, we employed submonolayer biolasers to detect α-syn in phosphate buffered saline (PBS). The initial surface density of Cy3 molecules was fixed at the threshold density so that lasing just above the laser threshold could occur. Note that α-syn detection operates in positive slope mode, while avidin detection operates in negative slope mode. The laser can achieve ultimate sensitivity with sparse conjugation of gain molecules near the threshold density, as predicted by numerical simulations (Fig. 2c). Precoating a fraction of Cy3 molecules before immunoassay reduces the number of antigen-affiliated gain molecules necessary to reach the laser threshold. The response of the laser emission to the biomarker concentration was calibrated by utilizing statistical analysis (Fig. S10), indicating an increase in the laser intensity with increasing biomarker concentration. The broadening of the statistical distribution was probably caused by the nonspecific binding at higher α-syn concentrations.

Then, we calibrated the sensing performance of submonolayer biolasers by detecting α-syn in human serum. Immunoassays in serum are frequently used for in vitro diagnostics but are more difficult than those in buffer due to the interference from complex components in serum. To enable disposable tests, fiber-supported submonolayer biolasers can be manufactured in batches (Fig. 5b). Same as the results in buffer, the statistical distribution in serum shifts towards higher laser intensity with a higher α-syn concentration (Fig. 5c). Figure 5d illustrates the calibration curves obtained in PBS and serum, both of which show good linearity of over *R*^2^ = 98% on the semi-log scale. The lower LODs in buffer and serum were estimated to be 0.43 pM (6.5 pg·ml^−1^) and 0.32 pM (4.8 pg·ml^−1^), respectively. This result is about three orders of magnitude lower than the α-syn in human serum of Parkinson’s disease patients^54^. The LOD in serum is slightly lower than that in PBS because the nonspecific binding of serum components on optical fiber blocks part of the binding sites of the capture antibody, lowering the intensity fluctuations. The lower intensity and narrower statistical distribution in serum than in buffer can also be attributed to the nonspecific binding phenomenon. High specificity was confirmed experimentally for the submonolayer laser biosensor (Fig. S11). Because of the high sensitivity and great specificity, the submonolayer biolaser is a potential candidate for clinic diagnosis.

Compared with the avidin detection experiment in Fig. 4, the calibration curve in Fig. 5d shows a larger dynamic range. The discrepancy in the dynamic range is due to the differences in molecular affinity. In general, the dissociation constant, which is inversely proportional to affinity, is used to characterize molecular affinity. With a dissociation constant of *K*_*d*_ ≈ 10^−15^ M, the avidin-biotin complex is one of the strongest known non-covalent bonds^55^. The dissociation constant for immunoglobulin G (IgG) antibody used in immunoassays typically ranges from 10^−9^ to 10^−12^, indicating a lower affinity than avidin-biotin binding (Fig. 4)^56^. According to the schematic illustrations in Fig. 5, the surface density of gain molecules, *S*, is in proportion to the concentration of analytes. The difference in surface density caused by the changes of analyte concentration can be expressed as *∆S* = *a∆C*, with *∆C* denoting the change of the analyte concentration. *a* is a constant that is significantly dependent on the molecular affinity. Given the same concentration change, the binding sites in the immunoreaction (α-syn detection in Fig. 5) will be less consumed than that in the avidin-biotin reaction (avidin detection in Fig. 3), resulting in a larger sensing dynamic range in Fig. 5.

## Discussion

We developed disposable submonolayer biolasers with ultrahigh sensitivity. Telecom optical fibers were utilized to provide continuously distributed optical microcavities with an ultrahigh Q-factor of 10^6^, resulting in a considerable reduction in the number of gain molecules required for lasing. The concept of a submonolayer biolaser was confirmed by observing the transition from stable monolayer biolasers to gradient submonolayer biolasers. We demonstrated that the ultimate sensitivity can be achieved by reducing the gain molecules to the threshold surface density. Ultrasensitive protein detection was achieved owing to the greatly enhanced light-matter interaction induced by the optical resonance in the microcavity and the laser amplification. In addition, mass production of this laser-based biosensor was demonstrated, e.g., over one million biosensors (~3 cm) can be fabricated in batches from a commercial spool of (50 km) telecom optical fibers. The good reproducibility of the biolasers and their biosensing was experimentally verified, which enables their disposable use. Ultrasensitive detection of a PD biomarker in buffer and serum was demonstrated, offering a general technique for early diagnosis of major diseases.

The high performance of submonolayer biolaser will help clinical applications in two ways. Firstly, a lower dilution rate might be used, which can result in less non-specific binding. Dilution was widely employed in real-world applications to reduce the non-specific binding effect of complex components in samples. As a tradeoff between sensor performance and the influence of non-specific binding, the dilution rate in the serum test was commonly fixed at 10%. Thanks to the outstanding sensing performance of submonolayer biolasers, a lower dilution rate of 1% can be applied. Since the α-syn concentration in the serum of Parkinson’s disease patients ranges from 300 pM to 1.7 nM^54^, the concentration of α-syn in the diluted serum is well within the linear detection range of submonolayer biolasers. Secondly, massive screening of diseases can be realized by applying pooled sample testing, which involves mixing several samples from different patients together in a pooled sample. This approach increases the number of individuals in the test, thus greatly boosting the time efficiency. However, because the samples are diluted, highly sensitive biosensors are required. Theoretically, the submonolayer biolasers enable pooled sample testing for 10 people, with a diagnostic efficiency boosted by 10 times (assuming a dilution rate of 1% was applied for the serum test). We envision that the submonolayer biolaser can potentially be applied for healthcare applications in clinics or hospitals.

Even though the whispering gallery mode providing optical feedback for lasing is temperature sensitive, its influence is negligible when the intensity interrogation was employed for sensing. In general, due to the phase change induced by temperature, the lasing peaks shifts while the integrated lasing intensity remains stable^57^. In addition, all the experiments were carried out at room temperature with no significant temperature changes. Because all the experiments were performed in a single-pulse pump mode and the pump laser scans along the fiber axis with a step of 250 μm after each pulse, the temperature variation caused by the pulsed pump laser can also be ignored.

## Materials and methods

### Experimental setup

A telecom single-mode optical fiber (Corning, SMF-28e) was exploited by using its silica cladding boundary as a continuously distributed microring resonator (Fig. 1a). As the refractive index of the optical fiber is higher than that of water, the optical fiber supports WGMs at the silica-liquid interface, providing optical feedback for lasing. The silica microresonators have low roughness owing to the melt drawing process and facilitate a high Q-factor for optical resonance. The details of the experimental setup are illustrated in Fig. S12. A pulsed laser (Continuum, 532 nm, 5 ns pulse width) was focused by a cylindrical lens into a pump strip of 150 μm × 5 mm. The pump energy density was kept at 1.5 mJ·mm^−2^ with the pump strip perpendicular to the fiber axis. To eliminate the dye molecule photobleaching effect, we scanned the pump along the fiber axis with a step of 250 μm and employed a single-pulse pump at each location. The emission spectrum was recorded by a diffraction grating spectrometer (Andor, SR500i) with a thermoelectrically cooled CCD (Andor, iDus 420 A). A blazed grating with 300 lines·mm^−1^ was used for spectral monitoring. A long-pass filter was used to eliminate the residual pump laser in the detection arm.

### Silanization of optical fibers

The polymer coating of the optical fibers was removed after immersing the fibers in acetone for 1 h. The bare optical fibers were hydroxylated in batches with freshly prepared piranha solution (a 3:7 volume mixture of 30% H_2_O_2_ and 98% concentrated H_2_SO_4_) overnight. Note that the effect of piranha solution can only last for 30 min. In order to maintain the activity of molecules, the following procedures were conducted in the next day and was preferred to be carried out continually. The hydroxylated optical fibers were cleaned three times by immersion in deionized (DI) water for 5 min each. After being washed in dry acetone for 20 min and dried in air, the optical fibers were silanized with (3-aminopropyl)triethoxysilane (APTES) (5% in acetone, v/v) for 6 h. The silanized optical fibers were cleaned in acetone, ethanol, and phosphate buffer (PBS, pH = 7.4) for 10 min each. Then, the silanized optical fibers were immersed in PBS and were ready for further experiments.

### Fabrication of submonolayer biolasers

An N-hydroxysuccinimide (NHS)-biotin stock solution with a concentration of 29 mM was prepared by dissolving lyophilized powder (Aladdin, N103916) in dimethyl sulfoxide (DMSO). The working solution was freshly prepared by diluting the stock solution in PBS. The working solution with a concentration of 200 μM was used for the experiments in Fig. 3a–c, while a lower concentration of 32 μM was used for exploring the ultimate sensitivity of the submonolayer biolasers in Fig. 4 (yellow curve). The silanized optical fibers were incubated in the NHS-biotin working solution for 30 min. After being washed three times in PBS for 10 min each, the biotinylated optical fibers were immersed in avidin solutions with various concentrations (0 pM, 0.1 pM, 1 pM, 10 pM, 100 pM, and 1000 pM) for 20 min. After being washed, the optical fibers were incubated in 100 μg·ml^−1^ Sav-Cy3 (Sigma, No. S6402) in PBS for 40 min to enable conjugation between Sav-Cy3 and biotin molecules on the optical fiber. After being washed three times with wash buffer (0.05% Tween 20 in PBS, v/v) for 10 min each, the optical fibers were immersed in PBS for the laser experiment.

### Fabrication of monolayer biolasers

The process of fabricating a monolayer biolaser was the same as that for the submonolayer biolaser, except that a higher concentration of the NHS-biotin working solution (1000 μM) was used for biotinylation.

### Fabrication of multilayer biolasers

The multilayer biolasers were fabricated by conjugating multiple layers of Cy3 molecules on the monolayer biolasers. The monolayer biolasers were immersed in DMSO for 5 min to remove water molecules on the fiber surface and were treated with the NHS-biotin working solution (1000 μM) for 30 min. This step was applied to introduce biotin molecules onto streptavidin molecules. Then, the NHS-biotin-treated monolayer biolasers were immersed in 100 μg·ml^−1^ Sav-Cy3 in PBS for 40 min. The NHS-biotin and Sav-Cy3 treatments were repeated five times to enable five-layer conjugation of Cy3 molecules. After being washed three times with wash buffer for 10 min each, the optical fibers were immersed in PBS for the laser experiment.

### Threshold characterization in single-use mode

The typical laser threshold curve is shown in Fig. S3. Each point was obtained by pumping one location on the optical fiber with a single pulse and then updating the optical fiber location by a scanning step of 250 μm.

### Q-factor measurement

The bare optical fibers were treated with piranha solution overnight, followed by washing with DI water. Then, the optical fibers were immersed in PBS for Q-factor measurements (Fig. S2a). An optical spectrum analyser with ultrahigh spectral resolution was constructed by a narrow-linewidth tuneable laser (New Focus, Model TLB6704-P), a photodetector (New Focus, Model 1801), and a digital oscilloscope (YOKOGAWA, Model DLM3034). The tuneable laser was coupled into and out of the optical fiber microcavity through a fiber taper, which was aligned perpendicularly to the optical fiber and finely adjusted through five-dimensional translation stages.

### Microscopic characterization of the biomolecular film

A fluorescence image of a submonolayer biolaser was obtained by a laser confocal microscope (A1R MP^+^, Nikon), which was equipped with a 20× water immersion objective lens and an excitation wavelength of 561 nm.

### Quantification of the laser intensity

We calculated the spectral integral of the intensity by using $I={\int }_{a}^{b}i\left(\lambda \right)d\lambda$. Here, *i*(*λ*) denotes the spectral distribution of the laser emission. [a, b] defines the spectral range of the laser emission. Then, the spectral integral of the intensity was converted into decibels by using ${I}_{{dB}}=10{\log }_{10}\left(I\right)$.

### Single use test

Six segments of optical fibers randomly selected from a 10 km spool were used for single use test. For each segment of fiber, the pump laser was scanned along the fiber axis with a step of 250 μm, and the laser spectrum at each location was recorded. The statistical result of the submonolayer laser emission is shown in Fig. 2e. Then, we calculated the average intensity of the *i*th optical fiber, which is denoted *I*_*i*_. The intensity variation of different optical fibers was calculated by using ${\rm{\delta }}={\rm{\sigma }}/\bar{I}$. Here, *σ* is the standard deviation, and $\bar{I}$ is the mean value of ${I}_{i}\left(i=\mathrm{1,2},\ldots 6\right)$.

### Immunoassay with submonolayer biolasers

A conceptual illustration of the α-syn immunoassay is given in Fig. 5a. The silanized optical fibers were treated with the NHS-biotin working solution (32 μM) for 30 min and immersed in DMSO for 5 min to remove water molecules on the fiber surface. The optical fibers were further treated with 50 mg·ml^−1^ disuccinimidyl substrate (DSS) in DMSO for 2 h. After being washed in DMSO for 10 min to remove the residual DSS molecules, the optical fibers were incubated in 120 μg·ml^−1^ of the capture antibody in PBS for 2 h. This was followed by three 5 min washes with wash buffer, which was exploited for the subsequent wash processes after each incubation. Then, the optical fibers were treated with Sav-Cy3 (100 μg·ml^−1^ in PBS) for 40 min to add a group of background gain molecules. The optical fibers were immersed in the blocking buffer (0.25% bovine serum albumin (BSA) in PBS, R&D Systems, DY995) for 1 h. After 5 min of washing three times with wash buffer, the optical fibers were immersed in PBS and were ready for immunoassay.

For the PD biomarker assay, the buffer solution (1% BSA in PBS) was freshly prepared by diluting the reagent diluent concentrate (×10) with DI water. The analyte solution was freshly prepared by diluting the α-syn stock solution to the desired concentration in the buffer solution. For PD biomarker detection in serum, the serum (Xinfan Biotechnology, No. 20211119) was diluted 10 times with PBS. Then, the analyte solution was freshly prepared by diluting the α-syn stock solution with serum. The optical fibers were incubated in α-syn at different concentrations for 1 h. The optical fibers were then immersed in 1.5 μg·ml^−1^ of the detection antibody for 1 h. We employed 100 μg·ml^−1^ Sav-Cy3 in PBS to treat the optical fiber for 40 min. The submonolayer biolasers were immersed in PBS for further testing. The capture antibody, α-syn, and detection antibody used in our experiment were from a commercial ELISA kit (R&D Systems, No. DY1338).

### Calculating the limit of detection

The relative intensity in Fig. 5 shows a linear relationship with the α-syn concentration on the semi-log scale. Hence, the linear fitting of the calibration curve can be written as *Y* = *kX* + *b*, with *k* and *b* denoting the slope and intercept of the linear fitting, respectively. *X* = log_10_(*x*) is the common logarithm of the α-syn concentration (*x*). The LOD was calculated by using ${LOD}={10}^{(3{E}_{r}-b)/k}$. Here, ${E}_{r}=2{\times }\left(1.96{\times }{SD}/\sqrt{N}\right)$ defines the width of the 95% confidence interval for the blank control. *SD* denotes the standard deviation, and *N* denotes the number of data points.

### Supplementary information

Supplementary Information for: Submonolayer Biolasers for Ultrasensitive Biomarker Detection

## Data Availability

All data are available within the Article and Supplementary Files, or available from the corresponding authors on reasonable request.
