# Supplementary material for: Submonolayer biolasers for ultrasensitive biomarker detection
Source: Light Sci Appl. 2023 Dec 6;12:292. doi: 10.1038/s41377-023-01335-8 (PMC10698069; doi:10.1038/s41377-023-01335-8)
Supplement: Supplementary file 1 — Supplementary Information for: Submonolayer Biolasers for Ultrasensitive Biomarker Detection [file 41377_2023_1335_MOESM1_ESM.docx]

Supplementary Information for:

**Submonolayer Biolasers for Ultrasensitive Biomarker Detection**

Chaoyang Gong^1,3,8^, Xi Yang^1,2,8^, Shui-Jing Tang^2,8^, Qian-Qian Zhang^1,8^, Yanqiong Wang^1^, Yi-Ling Liu^1^, Yu-Cheng Chen^4^, Gang-Ding Peng^6^, Xudong Fan^7^, Yun-Feng Xiao^2,^*, Yun-Jiang Rao^1,5,^*, and Yuan Gong^1,^*

^1^ Key Laboratory of Optical Fiber Sensing and Communications (Ministry of Education of China), School of Information and Communication Engineering, University of Electronic Science and Technology of China, Chengdu, Sichuan 611731, China.

^2^ State Key Laboratory for Mesoscopic Physics and Frontiers Science Centre for Nano-optoelectronics, School of Physics, Peking University, Beijing 100871, China.

^3^ Key Laboratory of Optoelectronic Technology and Systems (Ministry of Education of China), School of Optoelectronic Engineering, Chongqing University, Chongqing 400044, China.

^4^ School of Electrical and Electronic Engineering, Nanyang Technological University, Singapore 639798, Singapore.

^5^ Research Centre for Optical Fiber Sensing, Zhejiang Laboratory, Hangzhou, Zhejiang 310000, China.

^6^ School of Electrical Engineering and Telecommunications, University of New South Wales, Sydney, NSW 2052, Australia.

^7^ Department of Biomedical Engineering, University of Michigan, Ann Arbor, Michigan 48109, USA.

^8^ These authors contributed equally: Chaoyang Gong, Xi Yang, Shui-Jing Tang, and Qian-Qian Zhang.

*E-mails: [yfxiao@pku.edu.cn](mailto:yfxiao@pku.edu.cn); [yjrao@uestc.edu.cn](mailto:yjrao@uestc.edu.cn); [ygong@uestc.edu.cn](mailto:ygong@uestc.edu.cn)

**CONTENTS**

[**1. Surface modification** 2](#_Toc147045117)

[**2. Q-factor measurement of the optical fiber microcavities** 2](#_Toc147045118)

[**3. Laser threshold** 2](#_Toc147045119)

[**4. Demonstration of laser emission** 3](#_Toc147045120)

[**5. Numerical simulation of optical resonance in the fiber microcavity** 3](#_Toc147045121)

[**6. Analysis of the surface density of Cy3 molecules** 4](#_Toc147045122)

[**7. Characterization of laser emission** 5](#_Toc147045123)

[**7.1 Reproducibility of laser threshold** 5](#_Toc147045124)

[**7.2 Statistical distribution of laser emission** 6](#_Toc147045125)

[**8. Ultrahigh sensitivity of the submonolayer biolasers** 6](#_Toc147045126)

[**8.1 Theoretical model for sensitivity analysis** 6](#_Toc147045127)

[**8.2 Exploring the ultimate sensitivity of the submonolayer biolasers** 7](#_Toc147045128)

[**9. Alpha-synuclein detection in buffer** 8](#_Toc147045129)

[**10. Specificity test of submonolayer biolasers** 8](#_Toc147045130)

[**11. Experimental setup** 9](#_Toc147045131)

**1. Surface modification**

The process for the surface modification of optical fiber is illustrated in Fig. S1a, including hydroxylation, silanization, biotinylation, as well as specific conjugation between biotin and streptavidin molecules. The details are also described in Materials and Methods.

According to the chemical structures of molecules, the molecular spacer arms are 0.33 nm^1^, 1.35 nm^2^, and 2-4 nm^3,4^ in length for APTES, NHS-biotin, streptavidin, respectively. Therefore, the total thickness can be approximately evaluated to be between 3.68 nm and 5.68 nm. The purpose of the evaluation of thickness is to confirm that all the gain molecules were within the reach of the evanescent wave of the optical fiber microcavities, about 100 nm according to the numerical simulation (Fig. S5).


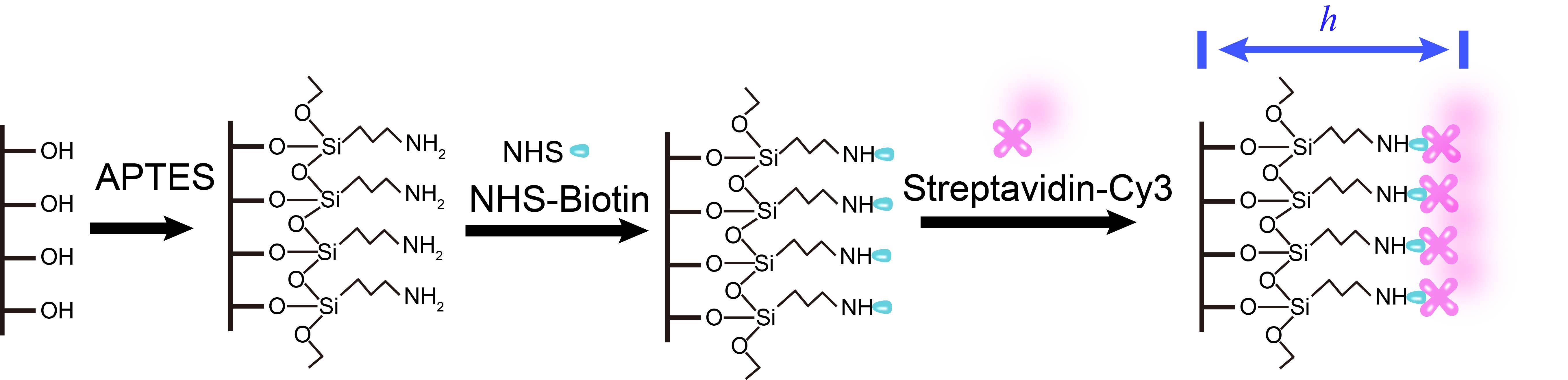


**Figure S1.** Illustration of the surface modification for the submonolayer biolaser based on the biotin-streptavidin conjugation. *h* denotes the distance from the Cy3 molecules to the surface of the silica optical fiber.

**2. Q-factor measurement of the optical fiber microcavit****ies**

The bare optical fibers were immersed in PBS, and the fiber taper coupling method illustrated in Fig. S2a was employed for Q-factor measurements. The detailed procedure is described in Materials and Methods. We randomly selected 22 different locations on the optical fiber to test. A typical transmission spectrum is shown in Fig. S2b. The average Q-factor of the optical fiber microcavities was measured to be 1.2×10^6^ with a coefficient of variation of 16 % (Fig. S2c).

**
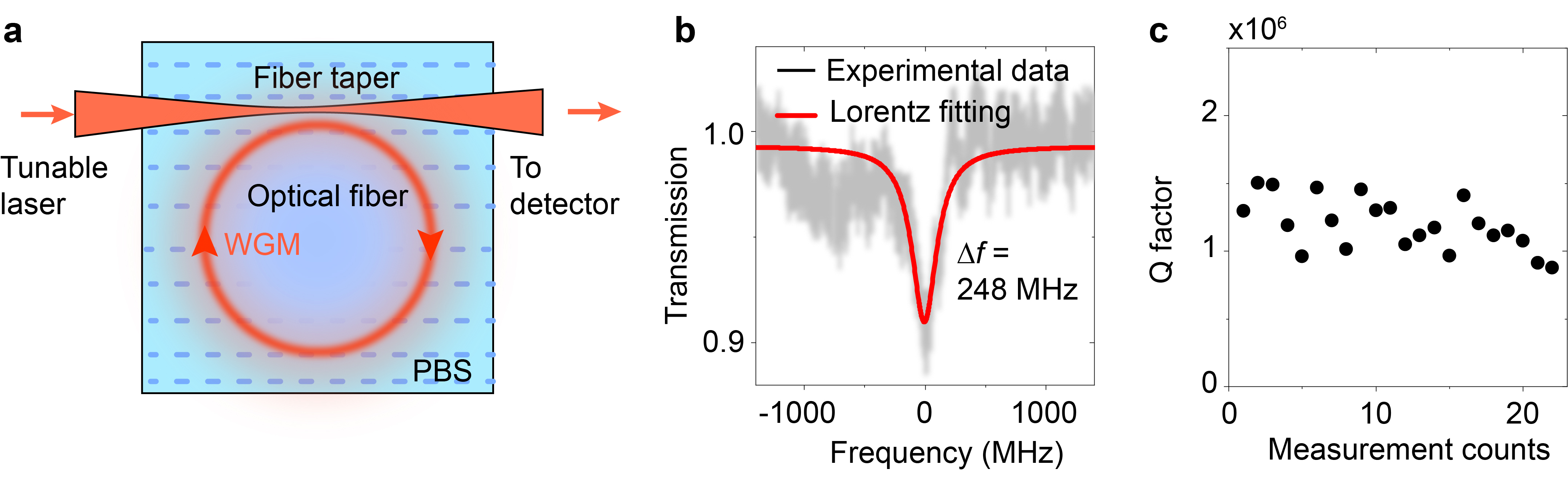
**

**Figure S2. a**, Schematic illustration of the experimental setup for Q-factor tests. **b,** A typical resonant dip in the frequency domain. **c,** The measured Q-factor of the microcavities at different locations of an optical fiber.

**3. Laser threshold**

The laser threshold of the submonolayer biolaser is strongly dependent on the surface density of gain molecules, which can be controlled with biotin concentrations. The typical laser threshold curves with different biotin concentrations are illustrated in Fig. S3.


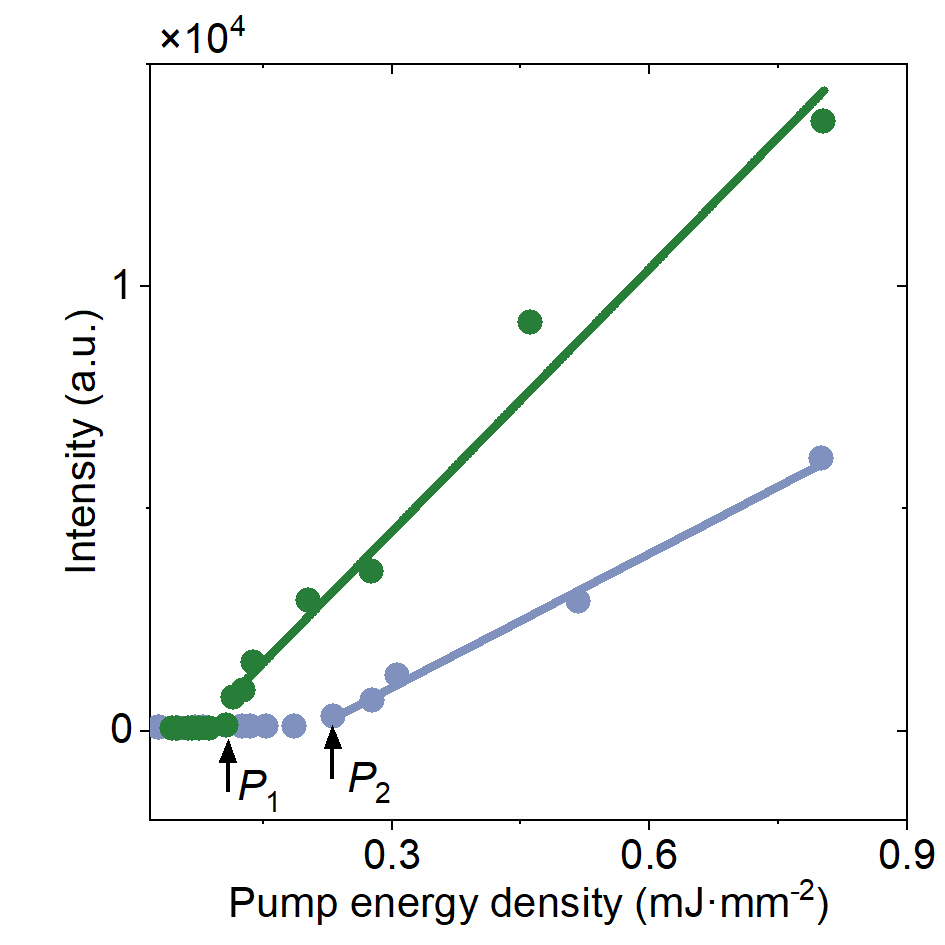


**Figure S3.** The threshold curves with biotin concentrations of 400 μM (green) and 200 μM (blue), respectively. Laser thresholds: *P*_1_ = 0.09 mJ⋅mm^-2^; *P*_2_ = 0.22 mJ⋅mm^-2^.

**4. Demonstration of laser emission**

We monitored the linewidth and intensity revolution by gradually increasing the pump energy density to demonstrate laser emission. To further resolve the spectrum, the spectrometer resolution was set to 0.11 nm. As illustrated in Fig. S4, the emission intensity shows a clear threshold behavior near 0.25 mJ⋅mm^-2^. Meanwhile, the linewidth started to decrease near the threshold pump energy density. The dynamic range of linewidth evolution is limited by the resolution of spectrometer. This result indicates the occurrence of lasing action^5-7.^


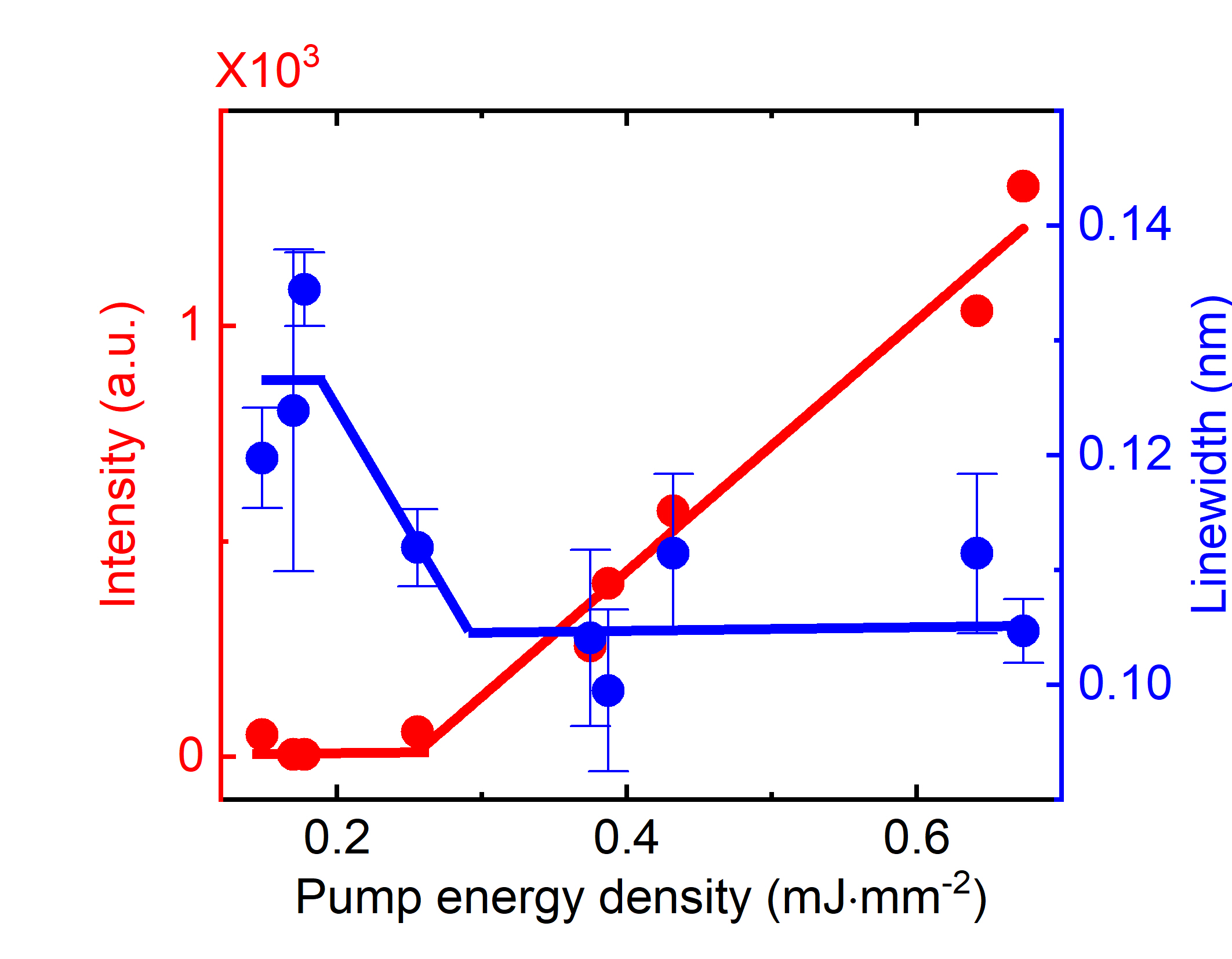


**Figure S4**. Light-light curve of the submonolayer biolaser and the linewidth evolution curve under varied pump energy density.

**5. Numerical simulation of optical resonance in the fiber microcavity**

We use finite element method to simulate the electric-field distribution of the optical fiber microcavity. The model consists of a two-dimensional ring cavity with a diameter of 125 μm. The main material property coefficients used in the simulation are the refractive index of the silica cavity (*n*_si_ = 1.45) and liquid medium (*n*_liquid_ = 1.33). Furthermore, a scattering boundary condition was set in the simulation. The numerical simulations show the optical resonance at specific resonant frequencies in the optical fiber microcavity (Fig. S5a). The enlargement of the intensity distribution in Fig. S5b indicates a penetration depth of about 100 nm of the evanescent wave (inset of Fig. S5b).


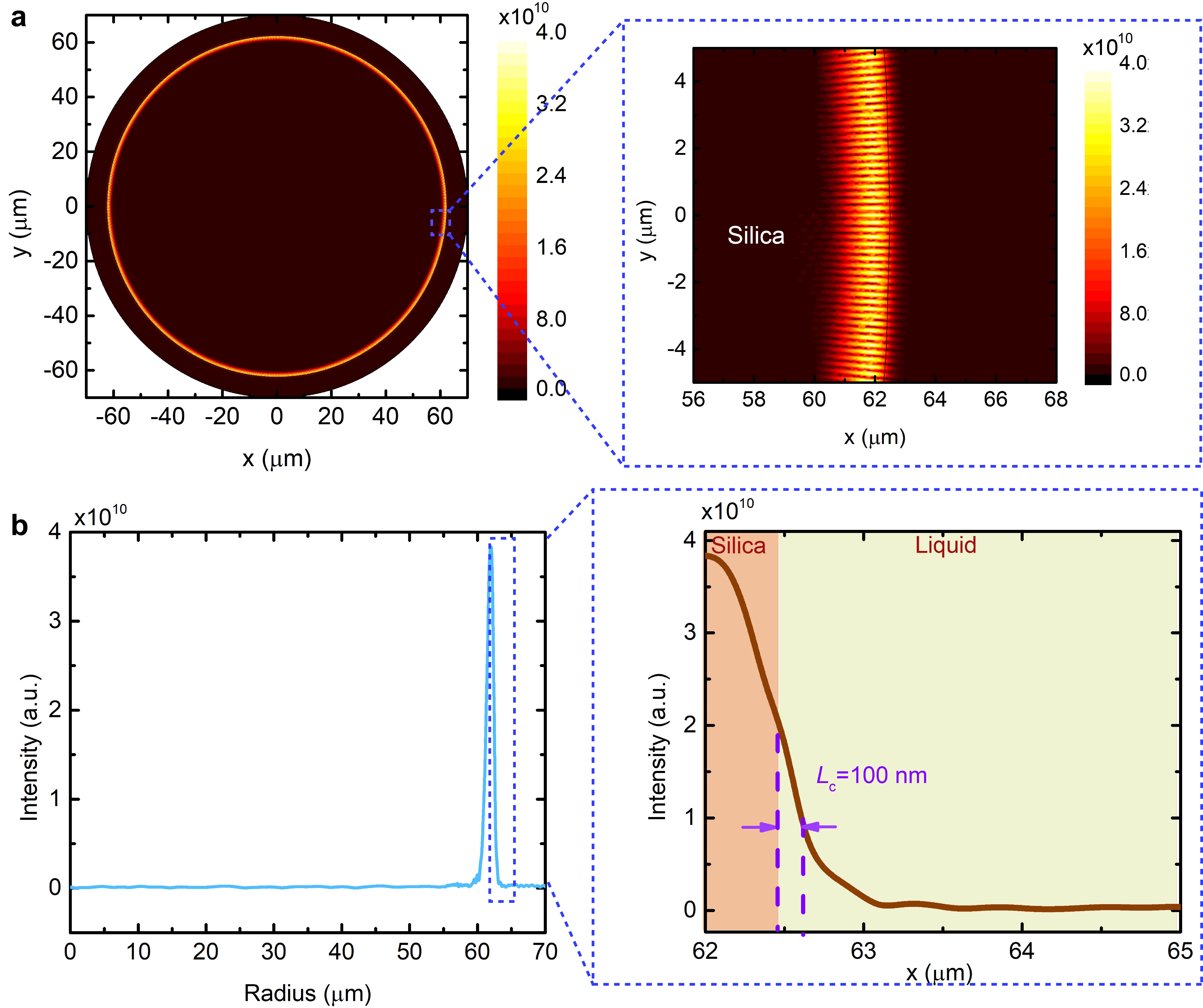


**Figure S5.** **a**, Numerical simulation of intensity distribution on the cross-section of the optical fiber. Inset, enlargement of the region in the dashed box. **b,** The radial intensity distribution with a resonant peak, data was derived from **a**. Inset, enlargement of the boxed region.

**6. Analysis of** **the surface density of Cy3 molecules**

The threshold condition for a four-level laser system can be expressed as^8^

$\eta D_{1}\sigma_{e}\left( \lambda\right)=\eta\sigma_{a}\left( \lambda\right)D_{0}+\frac{2\pi n}{\lambda_{L}Q}$ (S1)

Here, $\eta$ = 2.67% is the fraction of energy that interacts with the gain molecules, which can be calculated in Fig. S5. $D_{0}$ and $D_{1}$ are the density of dye molecules in the ground state and the lowest excited singlet state, respectively. $\sigma_{e}$ and $\sigma_{a}$ represent the emission and absorption cross-sections, respectively. $\lambda_{L}$ denotes the laser wavelength and $Q$stands for the Q-factor. $n$ is the effective refractive index of the exciting mode of the fiber ring resonator. Thus, the fraction of molecules in the excited state can be given by

$\gamma=\frac{D_{1}}{D_{0}+D_{1}}=\frac{\sigma_{a}\left( \lambda\right)}{\sigma_{e}\left( \lambda\right)}\left( 1+\frac{2\pi n}{\lambda_{L}D\eta Q\sigma_{a}\left( \lambda\right)} \right)$ (S2)

Here, $D=D_{0}+D_{1}$ denotes the density of total gain molecules. According to the rate equation for a four-level laser system, $\gamma$ can also be approximated as^9^

$\gamma=\frac{w_{p}\tau_{rad}}{1+w_{p}\tau_{rad}}$ (S3)

Here, $w_{p}={I_{th}\sigma_{a}}/{E_{0}\Delta t}$ is the normalized pump intensity. $I_{th}$ is the laser threshold. $E_{0}={hc}/{\lambda_{p}}$ is the photon energy of the pump. $\Delta t$ is the pulse width of the pump laser. $\tau_{rad}$ is the lifetime of the excited state.

According to the conclusions in Figs. S1 and S6, all the Cy3 molecules conjugated on the optical fiber are located within the reach of the evanescent field and can thus be involved in the optical resonance. Therefore, once the laser threshold is measured, *N* can be numerically calculated by using Eqs. S2 and S3. The surface density of gain molecules is calculated using *S*=*D***d*, with *d* =100 nm denoting the penetration depth (Fig. S5). We calculated the dependence of $I_{th}$ on *S* with various Q-factors and the results are illustrated in Fig. S6, through which we can estimate the surface density of gain molecules on fiber by the given threshold.


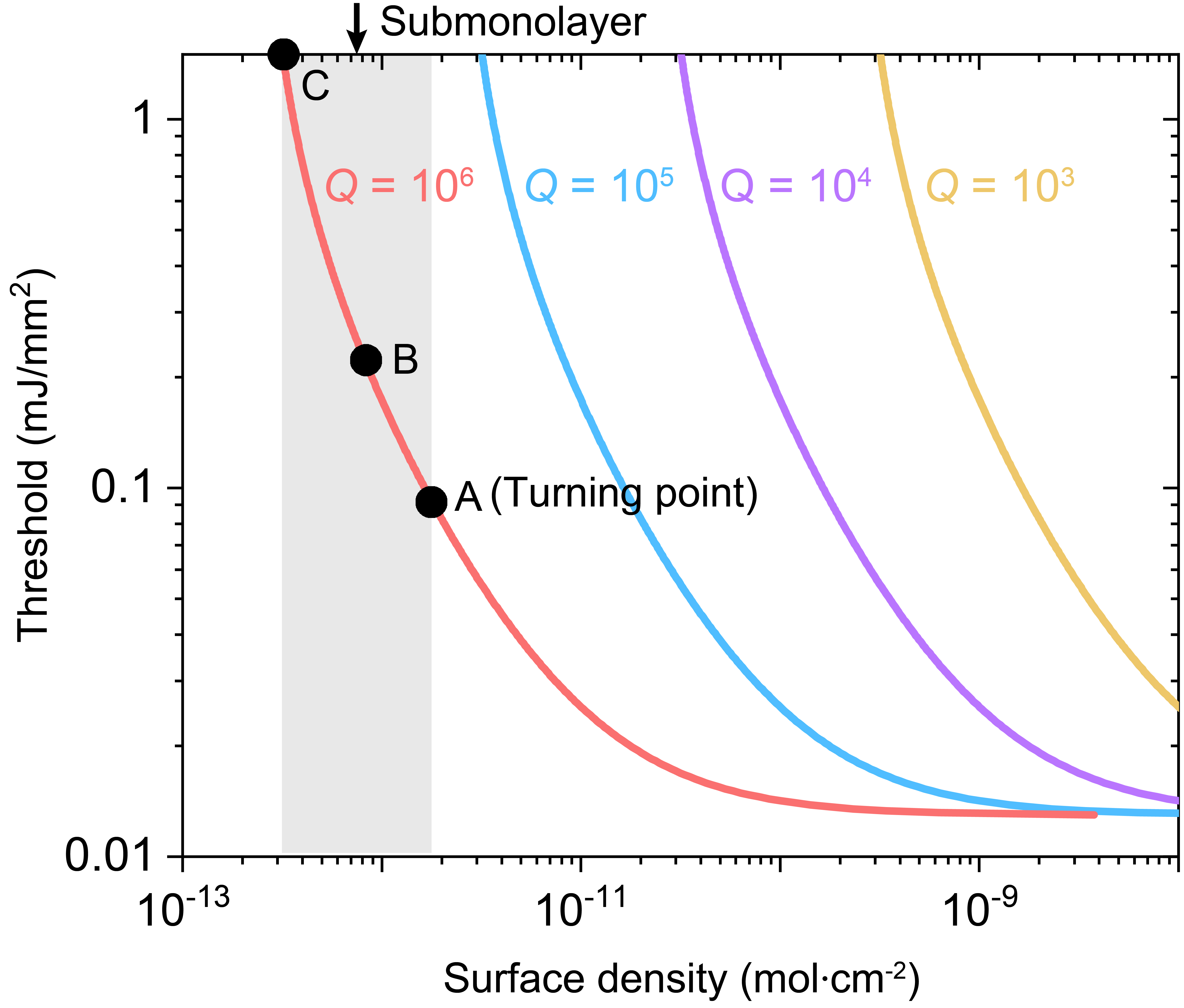


**Figure S6.** Numerical results of the laser threshold as a function of the surface density of Cy3 molecules. The grey area denotes the submonolayer biolaser.

**7. Characterization of laser emission**

**7.1 Reproducibility of laser threshold**

The frequency histogram of laser threshold was illustrated in Fig. S7, indicating an average threshold of 0.6 mJ⋅mm^-2^. Comparing with the threshold given in Fig. S3, the higher threshold is caused by a lower NHS-biotin concentration.


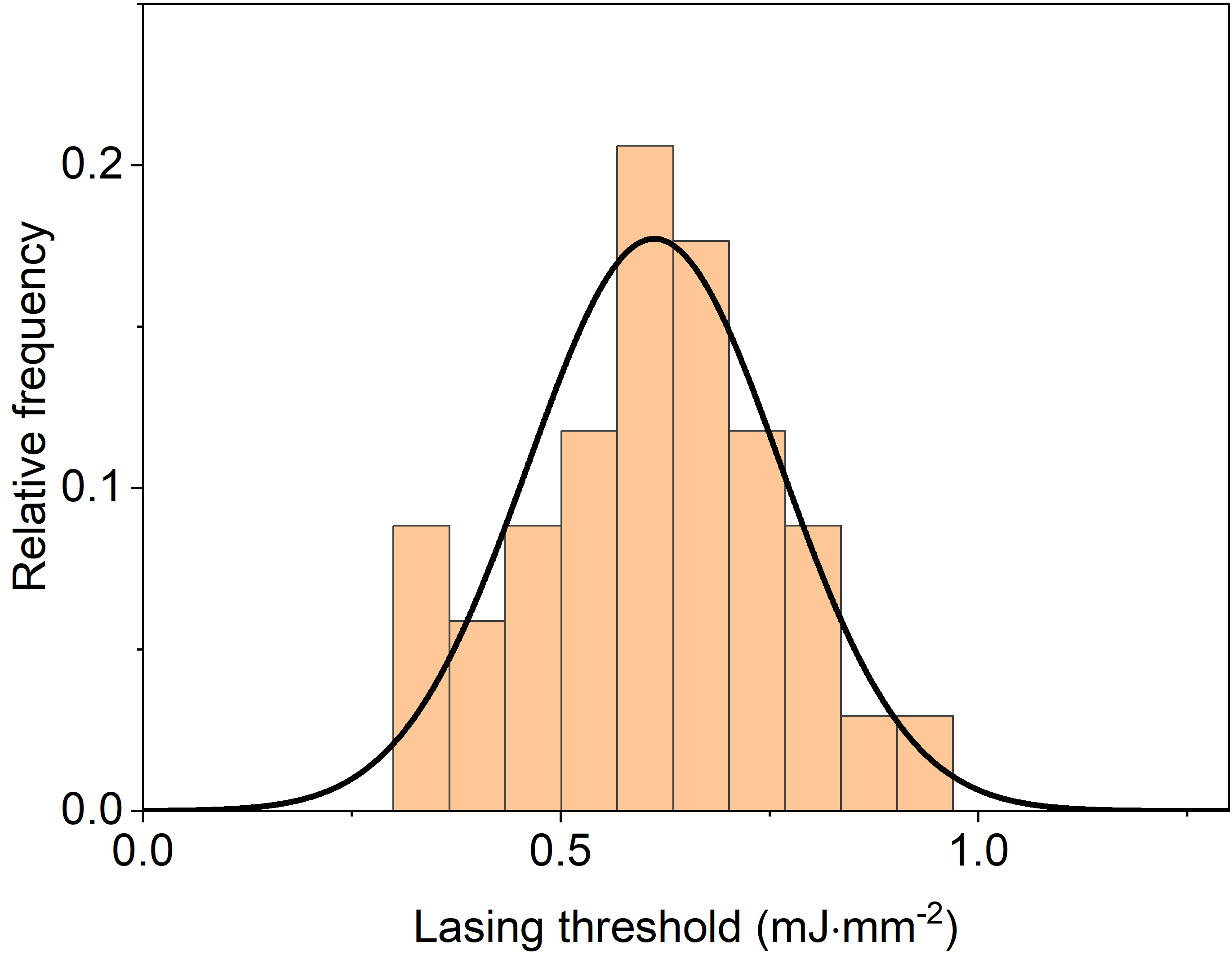


**Figure S7.** Frequency histogram of laser threshold. Data are extracted from 34 submonolayer biolasers. These submonolayer biolasers were fabricated using 100 μM NHS-biotin.

**7.2 Statistical distribution of laser emission**

A batch of 736 biolasers was tested to investigate the statistical distribution of the laser intensity (Fig. S8). The relative laser intensity in decibels of each test was calculated, which formed a normal distribution. The characteristics of the statistical distribution were analyzed and the mean was selected as a sensor indicator.


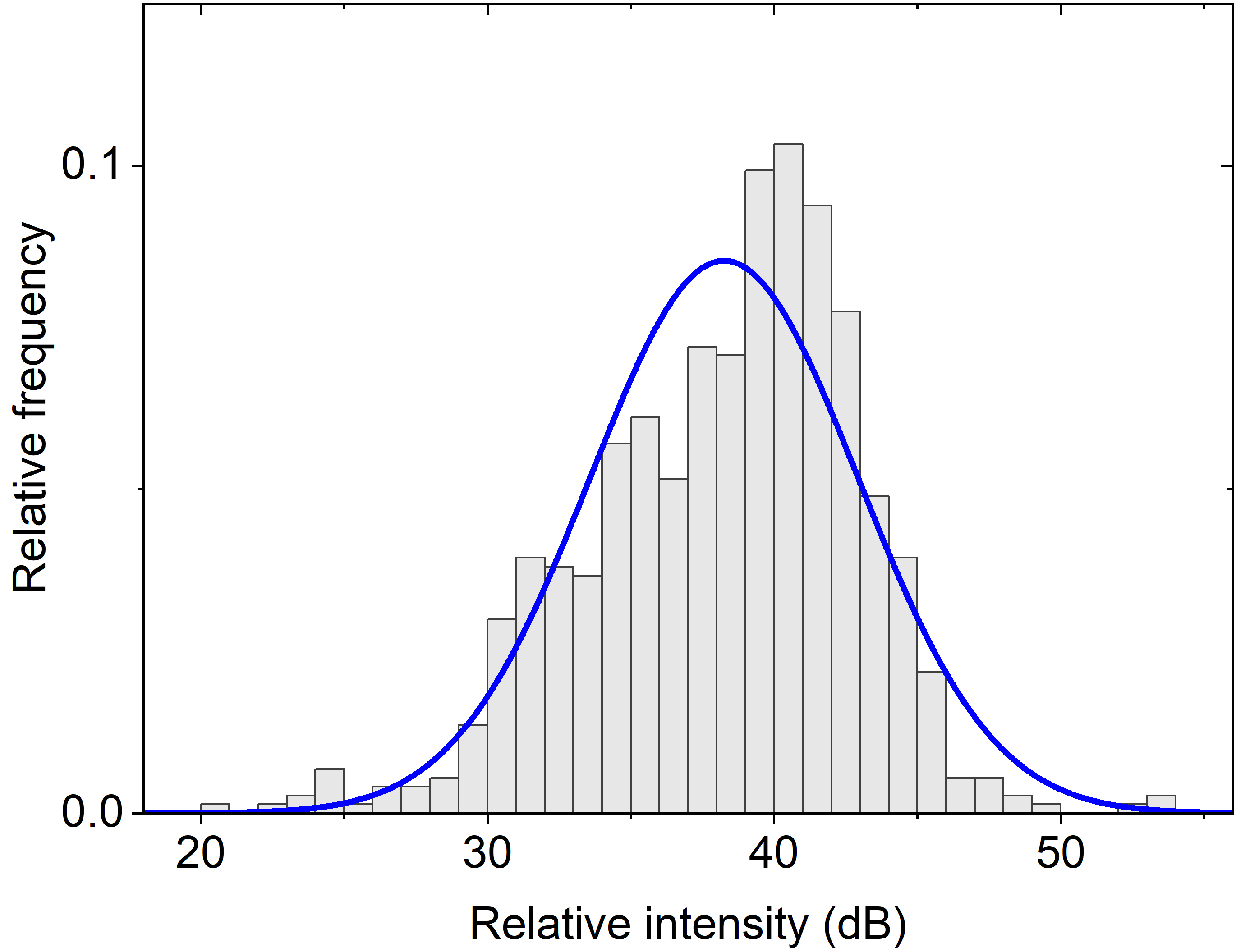


**Figure S8.** Frequency histogram of relative intensity. Data are extracted from 736 submonolayer biolasers.

**8. Ultrahigh sensitivity of the submonolayer biolasers**

**8.1 Theoretical model for sensitivity analysis**

The laser intensity can be written as^9^

$I_{laser}=A\left( \frac{I_{pump}}{I_{th}}-1 \right)$ (S4)

Here, $I_{pump}$ is the pump intensity and $I_{th}$ is the laser threshold. *A* is a constant.

Combining with Eqs. S3 to S4, we derive that

$I_{laser}=A\left[ \frac{I_{pump}{}_{a}\tau_{rad}}{E_{0}t}\left( \frac{1}{\gamma}-1 \right)-1 \right]$ (S5)

This equation shows the dependence of the laser intensity on the fraction of molecules in the excited state ($\gamma$). $\sigma_{a}$ represent the absorption cross-sections of gain molecules. $\tau_{rad}$ is the lifetime of the excited state. $E_{0}$ is the photon energy of the pump. $\Delta t$ is the pulse width of the pump laser. Then we can calculate the laser emission as a function of the surface density (*S*).

In immunoassay, the number of gain molecules is proportional to the number of conjugated antigen molecules. The sensitivity of the laser-based sensor to the antigen on fiber can be defined as

$\frac{dI_{laser}}{dS}=\frac{dI_{laser}}{d\gamma}\cdot\frac{d\gamma}{dD}\cdot\frac{dD}{dS}$ (S6)

With $\frac{dI_{laser}}{d\gamma}=-A\cdot\frac{I_{pump}\tau_{rad}\sigma_{a}}{E_{0}\Delta t}\cdot\frac{1}{\gamma^{2}}$ (S7)

$\frac{d\gamma}{dD}=-\frac{2\pi n}{\sigma_{e}\lambda_{L}\eta Q}\cdot\left( \frac{S}{d} \right)^{2}$ (S8)

and

$\frac{dD}{dS}=\frac{1}{d}$ (S9)

Here, $D$, *S* denotes the density and surface density of gain molecules, respectively. *d* is the penetration depth of evanescent wave. *n* is the effective refractive index of the exciting mode of the fiber ring resonator. $\sigma_{e}$ is the emission cross-sections of gain molecules. $\lambda_{L}$ denotes the laser wavelength and $Q$stands for the Q-factor. $\eta$ is the fraction of energy that interacts with the gain molecules.

According to the analysis above, theoretical calculation of the sensitivity as a function of Cy3 surface density was shown in Fig. 2c. The results indicate an increase in sensitivity with a lower surface density and an ultrahigh sensitivity can be achieved when the surface density decreases to the threshold density.

Comparatively, in the fluorescence tests, the emission intensity can be written as

$I_{FL}=B\cdot S{\cdot I}_{pump}$ (S10)

Here, $B$ is a constant. The sensitivity of the fluorescence-based sensor can be defined as

$\frac{{dI}_{FL}}{dS}=B{\cdot I}_{pump}$ (S11)

Therefore, the sensitivity of the fluorescence-based sensor remains independent of the molecular surface density.

**8.2 Exploring the ultimate sensitivity of the submonolayer biolasers**

The ultimate sensitivity can be achieved the surface density of gain molecules close to the threshold density (~ 3.2 ×10^-13^ mol⋅cm^-2^, Point C in Fig. S6). In this case, a dramatic decrease in lasing intensity can be observed when the avidin concentration reaches 1fM and the laser extinguished with 10 fM avidin. The typical emission spectra with various avidin concentrations are illustrated in Fig. S9.


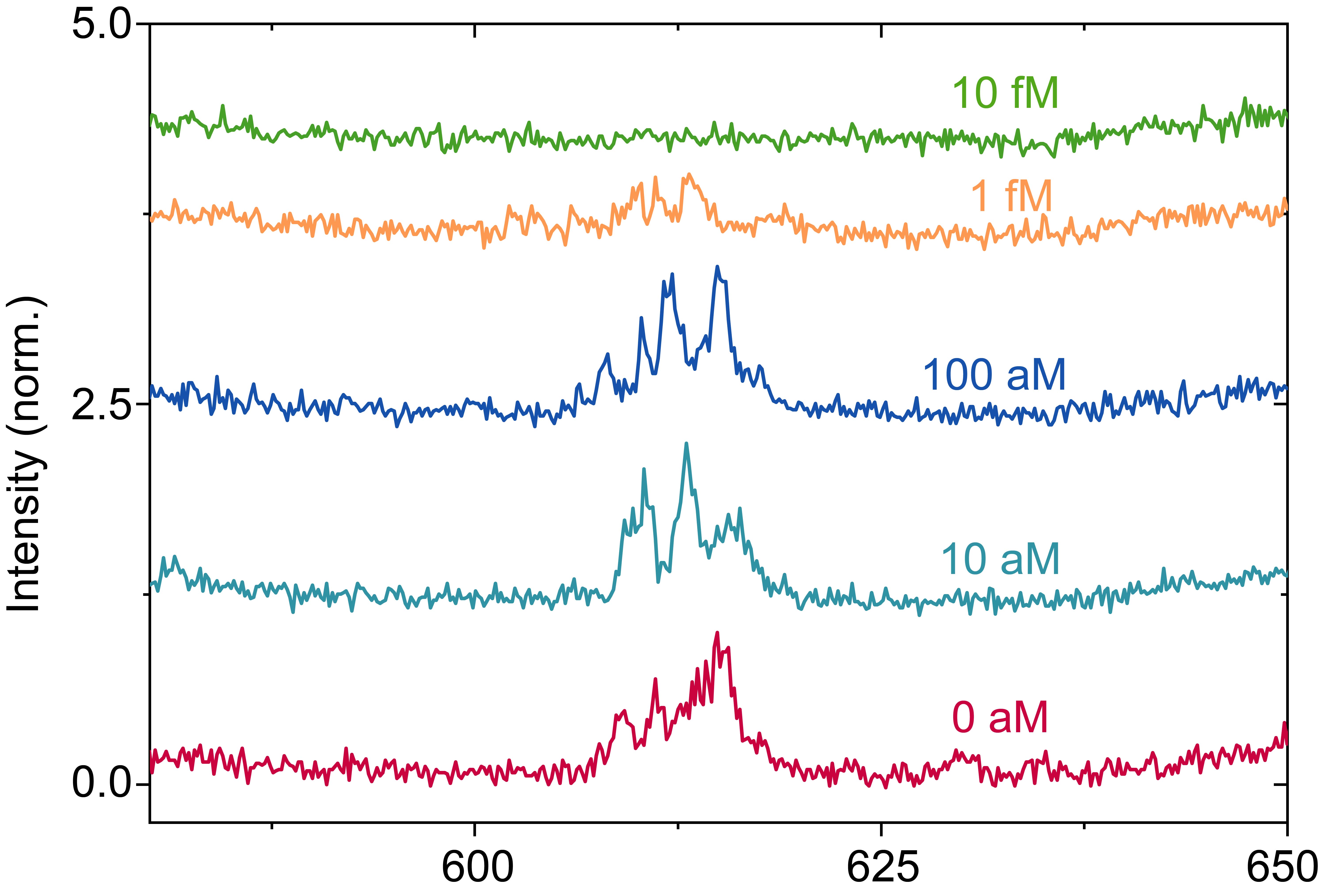


**Figure S9.** Spectral evolution of submonolayer biolaser with different concentrations of avidin.

**9. Alpha-synuclein detection in buffer**

We tested the performance of the submonolayer biolasers in buffer solution. The detailed procedure can be found in Materials and Methods. The statistical distribution continuously shifts toward right with a higher α-syn concentration (Fig. S10).


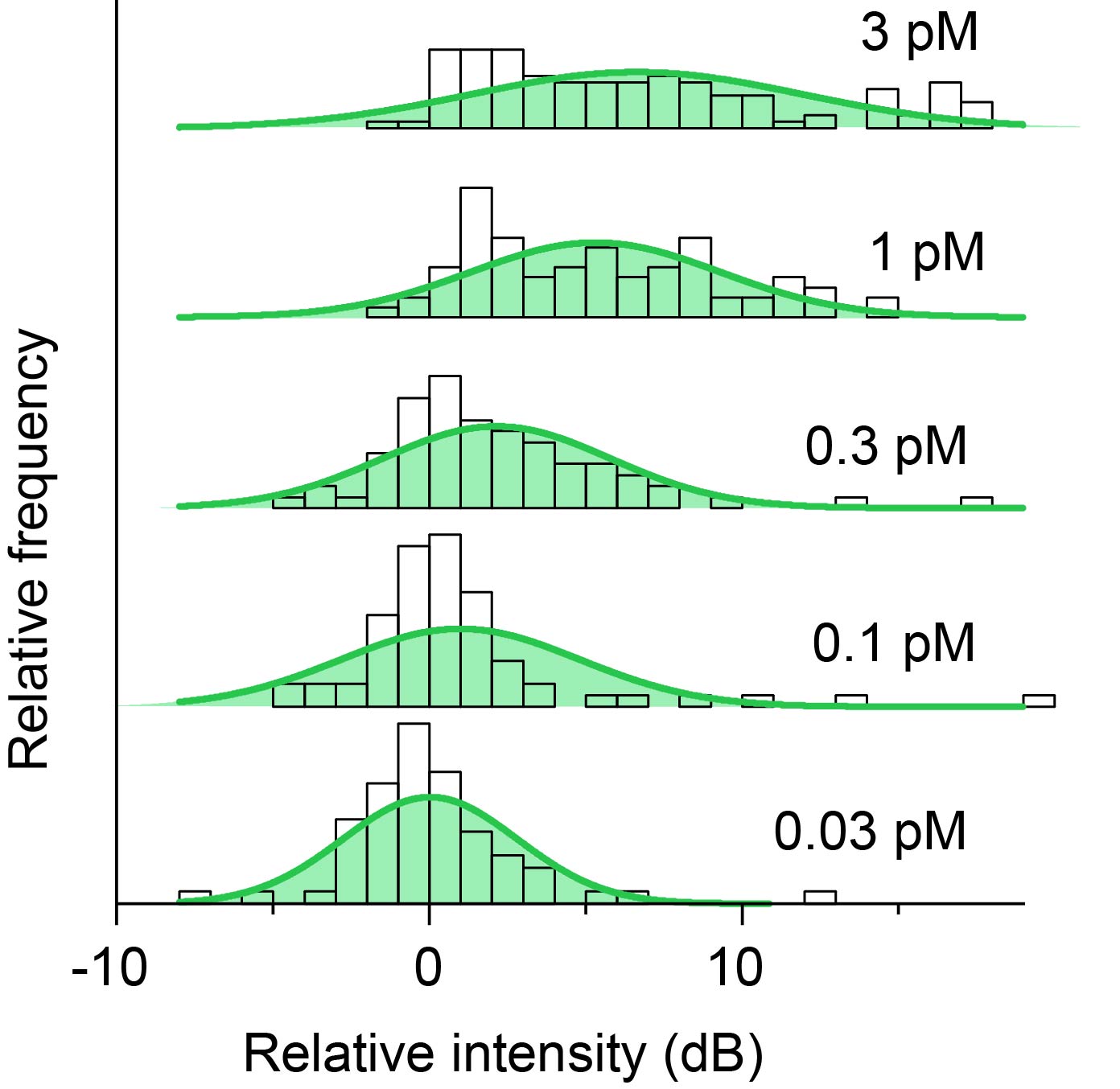


**Figure S10.** Statistical distribution of the relative intensity at different α-syn concentrations in buffer.

**10. Specificity test of submonolayer biolasers**

The specificity of submonolayer biolasers was tested in Fig. S11. Non-specific controls including Alzheimer's disease biomarkers such as amyloid beta 42 (Aβ42), amyloid beta 40 (Aβ42), and tumor biomarkers such as carcinoembryonic antigen (CEA), α-Fetoprotein (AFP), and neuron specific enolase (NSE) were evaluated. Meanwhile, two negative controls (without capture antibody and without a-syn) were examined. The result shows that only a-syn triggers a significant increase in laser intensity. Please be noted that the concentrations of the non-specific controls are at least ten times higher than in the physiological environment. This phenomenon confirms the great specificity of submonolayer biolasers.


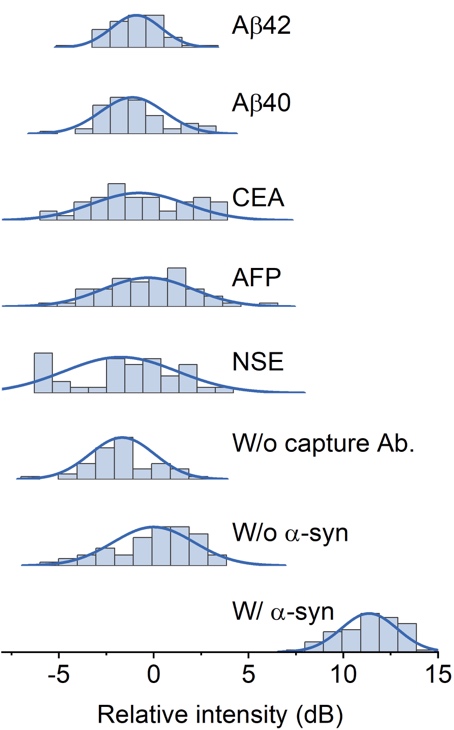


**Figure S11.** Specificity test of the submonolayer biolaser. Aβ42, 44.3 nM; Aβ40, 46.2 nM; CEA, 0.28 nM; AFP, 3.7 nM; NSE, 1.2 nM; α-syn, 200 pM.

**11. Experimental setup**

The experiment setup is illustrated in Fig. S12. The optical fiber was fixed on a motorized stage and the position of the optical fiber moves in the fiber axis direction with a step of 250 μm, thus enabling a quick scanning of the pump strip along the optical fiber. More details of the experimental setup can be found in Materials and Methods.


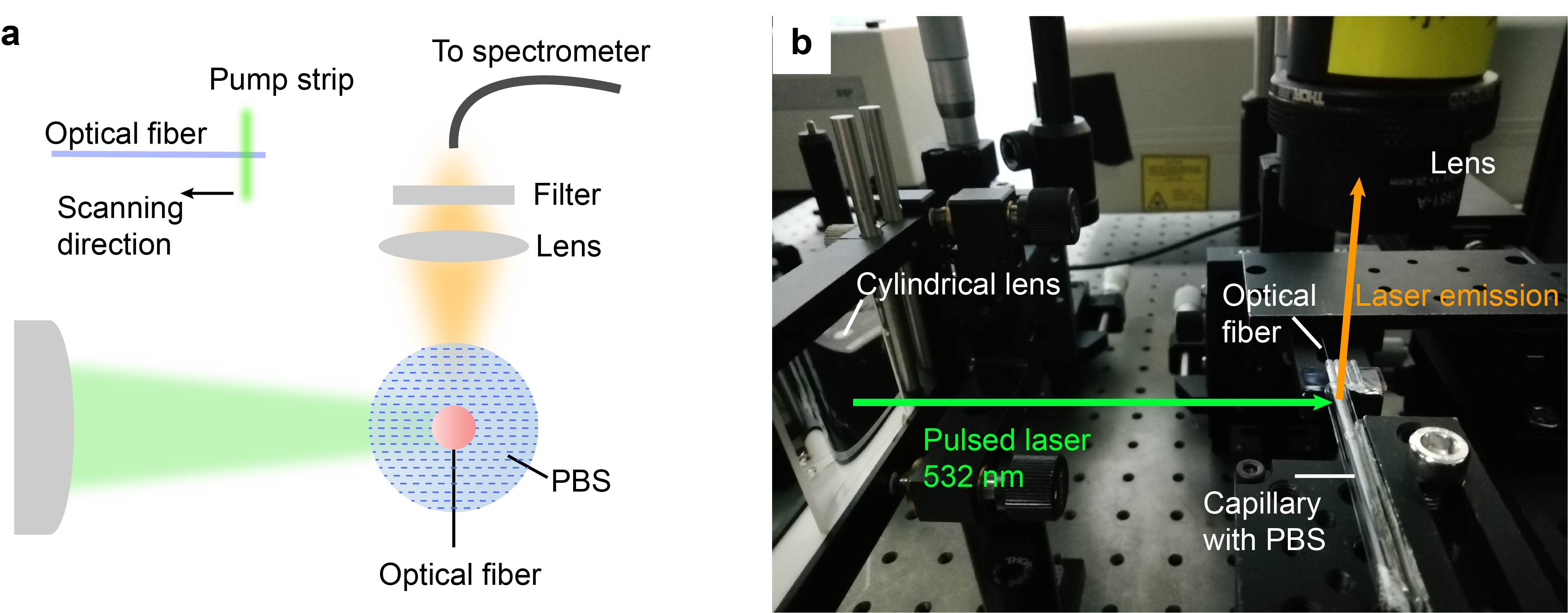


**Figure S12. a,** Conceptual illustration and **b**, the photo of the experimental setup.

**References**

1 Yang, S.-q. *et al.* Effect of reaction temperature on grafting of γ-aminopropyl triethoxysilane (APTES) onto kaolinite. *Appl. Clay Sci.* **62**, 8-14 (2012).

2 Cohn, J. V. *et al.* Extracellular lysines on the plasmodial surface anion channel involved in Na+ exclusion. *Mol. Biochem. Parasitol.* **132**, 27-34 (2003).

3 Ding, Z. *et al.* Size-dependent control of the binding of biotinylated proteins to streptavidin using a polymer shield. *Nature* **411**, 59-62 (2001).

4 Weber, P. C. *et al.* Structural origins of high-affinity biotin binding to streptavidin. *Science* **243**, 85-88 (1989).

5 Moon, B.-S. *et al.* Continuous-wave upconversion lasing with a sub-10 W cm−2 threshold enabled by atomic disorder in the host matrix. *Nat. Commun.* **12**, 4437 (2021).

6 Mao, X.-R. *et al.* Magic-angle lasers in nanostructured moiré superlattice. *Nat. Nanotechnol* **16**, 1099-1105 (2021).

7 Sang, Y.-G. *et al.* Topological polarization singular lasing with highly efficient radiation channel. *Nat. Commun.* **13**, 6485 (2022).

8 Moon, H.-J., Chough, Y.-T. & An, K. Cylindrical microcavity laser based on the evanescent-wave-coupled gain. *Phys. Rev. Lett.* **85**, 3161 (2000).

9 Siegman, A. E. *Lasers*. (University science books, 1986).
